# Diffuse Large B-Cell Lymphoma Arising from Cauda Equina: A Rare Case Report and Review of the Literature

**DOI:** 10.3390/diseases14040129

**Published:** 2026-04-02

**Authors:** Yuma Terada, Takafumi Yayama, Akira Nakamura, Kanji Mori, Narihito Kodama, Tomohiro Mimura, Kosei Ando, Kosuke Kumagai, Yoshinori Takemura, Shinji Imai

**Affiliations:** 1 Department of Orthopedic Surgery, Shiga University of Medical Science, Setatsukinowa, Otsu 520-2192, Shiga, Japan; yumaterada3526@gmail.com (Y.T.); kosei@belle.shiga-med.ac.jp (K.A.); kumamp@belle.shiga-med.ac.jp (K.K.); takemura@belle.shiga-med.ac.jp (Y.T.); simai@belle.shiga-med.ac.jp (S.I.); 2 Department of Orthopedic Surgery, Ohmihachiman Community Medical Center, 1379 Tsuchida, Ohmihachiman 523-0082, Shiga, Japan; akiranakamura1009@gmail.com (A.N.); koda62@belle.shiga-med.ac.jp (N.K.); 3 Department of Spine and Joint Reconstruction, Shiga University of Medical Science, Setatsukinowa, Otsu 520-2192, Shiga, Japan; kanchi@belle.shiga-med.ac.jp (K.M.); tmimura@belle.shiga-med.ac.jp (T.M.); 4 Department of Rehabilitation Medicine, Shiga University of Medical Science, Setatsukinowa, Otsu 520-2192, Shiga, Japan

**Keywords:** cauda equina lymphoma, non-Hodgkin lymphoma, diffuse large B-cell type, thoraco-lumbar spine

## Abstract

**Background:** Malignant lymphoma is the most common hematological malignancy; however, primary central nervous system lymphoma accounts for only a small percentage of non-Hodgkin lymphoma (NHL). Among these, primary cauda equina lymphoma (CEL) is extremely uncommon. Its rarity and atypical clinical presentation often make diagnosis challenging. **Case Presentation:** An 80-year-old man presented with progressive gait disturbance, lower-extremity weakness, and numbness. MRI revealed diffuse swelling and homogeneous gadolinium enhancement of the cauda equina at T12–L1; additionally, CSF cytology identified malignant lymphocytes. Open biopsy confirmed a diagnosis of diffuse large B-cell lymphoma. At diagnosis, the patient was classified as Ann Arbor stage IV, and the clinical parameters corresponded to a high-risk International Prognostic Index (IPI) category. The patient received five courses of immunochemotherapy with rituximab, methotrexate, vincristine, and procarbazine (R-MPV), resulting in marked radiological improvement and functional recovery, achieving a complete response. However, consolidation therapy was discontinued as the patient did not wish to continue. Unfortunately, intracranial relapse occurred four months later, and the patient ultimately succumbed to infectious complications. Only 29 cases of primary CEL have been reported. For all cases, a biopsy with histopathological examination is required for a definitive diagnosis. Currently, combined chemotherapy and radiotherapy are considered the standard treatment. This case was diagnosed through nerve biopsy with cauda equina at T12 to L1 levels, and immunochemotherapy successfully reduced the lesion while improving lower extremity function. **Conclusions:** Despite the considerable burden on patients, nerve biopsy is necessary for primary CEL to obtain a diagnosis and an early therapeutic approach for both neurological and vital prognoses.

## 1. Introduction

Non-Hodgkin lymphoma (NHL) invading the peripheral nerves, nerve roots, nerve plexuses, or cranial nerves is referred to as neurolymphomatosis (NL) [1]. CEL, classified within primary central nervous system lymphoma (PCNSL) in the updated 2022 WHO classification of hematolymphoid tumors (WHO-HAEM5), is an extremely rare entity, and its rarity and nonspecific clinical presentation often make early diagnosis difficult. To date, only 29 cases of primary CEL predominantly involving the cauda equina have been reported. The typical clinical features of NL include fever, weight loss, and site-specific neurological symptoms. These may include focal neurological deficits, neuropsychiatric symptoms, intracranial hypertension, and seizures [2,3]. Because these can mimic metastatic tumors, inflammatory conditions, or infectious diseases, diagnosis is often challenging, especially given the rarity of the disease [4].

We encountered a rare case of diffuse large B-cell lymphoma (DLBCL) arising from the cauda equina. In this study, we report on this case’s clinical and therapeutic course with the literature review of primary CEL.

## 2. Case Presentation

An 80-year-old man presented with gait disturbances due to motor palsy, lower back pain, and numbness in both extremities, without fever, night sweats, or weight loss. He had experienced progressive gait disturbance over the last 5 months without any obvious etiology. The patient had a history of Parkinson’s disease, hypertension, and type 2 diabetes. He was initially admitted to the Department of Neurology for suspected exacerbation of Parkinson’s disease-related symptoms, where medications were adjusted. Although his symptoms temporarily improved, the gait disturbances continued to worsen. The patient was then referred to the Department of Orthopedic Surgery. Physical examination during the initial orthopedic consultation revealed manual muscle testing (MMT) scores of 4/5 bilaterally in the iliopsoas and quadriceps muscles and 3/5 bilaterally in the tibialis anterior, extensor hallucis longus, and gastrocnemius muscles. Sensory examination demonstrated bilateral hypoesthesia across the lower extremities. Deep tendon reflexes in the lower extremities were normal, and the pathological reflex was negative. The patient required external support for ambulation.

**Figure 1:**
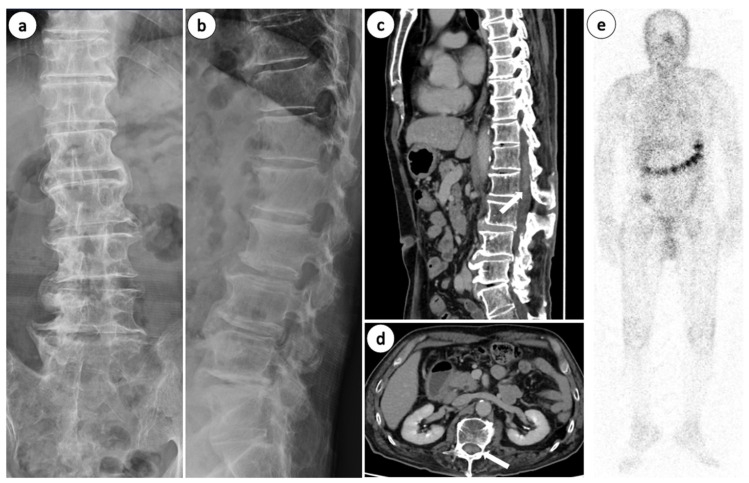
Plain lumbar spine radiographs in the (**a**) anteroposterior view and (**b**) lateral view. Contrast-enhanced CT showing the (**c**) sagittal section and (**d**) axial section at the L1 level. The arrow indicates the lesion. (**e**) Ga-scintigraphy. Radiographs and Ga-scintigraphy revealed no significant abnormalities. CT demonstrated enhancement of the cauda equina.

**Figure 2:**
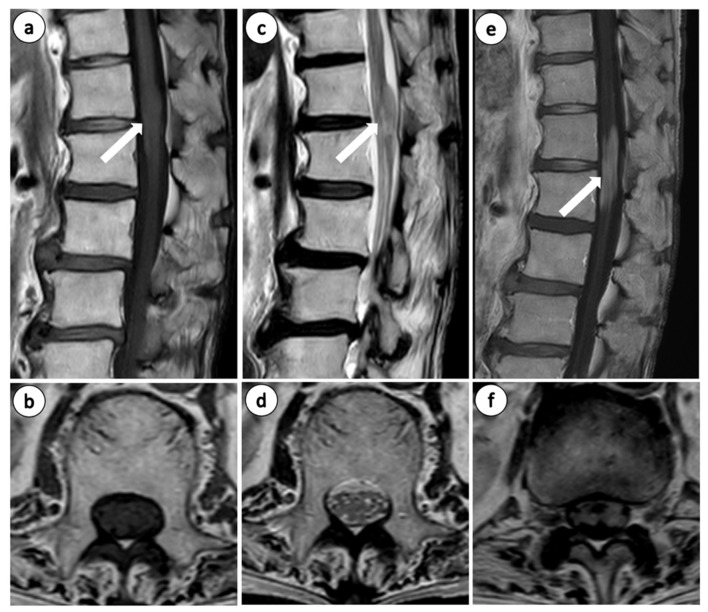
Lumbar MR images: T1WI (**a**) sagittal and (**b**) axial, and T2WI (**c**) sagittal and (**d**) axial section at the L1 level. Gadolinium-enhanced MR images: (**e**) sagittal and (**f**) axial section at the L1 level. The arrow indicates abnormal signal changes.

Laboratory investigations revealed a white blood cell (WBC) count of 5600/μL, with no abnormal lymphocytes detected in the peripheral blood smear. Additionally, lactate dehydrogenase (LDH) at 174 U/L and C-reactive protein (CRP) at 0.01 mg/dL were both within the normal reference ranges, and tumor markers (AFP, CA19-9, CEA, and PSA) and T-SPOT.TB were negative. Soluble interleukin-2 receptor (sIL-2R) was slightly elevated to 742 U/mL, suggesting lymphocyte activation which serves as a supportive biomarker for lymphoma. Urinalysis revealed trace proteinuria (±), and Bence Jones protein (BJP) was negative. Plain lumbar spine radiographs showed no significant abnormalities other than age-related changes (Figure 1a,b), and contrast-enhanced computed tomography (CT) of the chest, abdomen, and pelvis revealed enhancement of the cauda equina at the Th12–L1 level (Figure 1c,d); no other findings suggestive of neoplastic lesions were observed. No significant abnormal accumulation was observed on gallium scintigraphy of the whole body (Figure 1e). Lumbar magnetic resonance (MR) imaging showed abnormal signal intensity of the cauda equina extending approximately 4 cm in length at the Th12–L1 level, showing isointensity to hyperintensity signals on T1-weighted images and hypointensity signals on T2-weighted images compared to the spinal cord (Figure 2a–d). Gadolinium-enhanced T1-weighted MR revealed diffuse enhancement along the cauda equina at the same level (Figure 2e,f). Brain MRI revealed no nodular lesions. Cerebrospinal fluid (CSF) examination revealed a cell count of 110/μL, protein level of 197.2 mg/dL, and glucose level of 61 mg/dL. Small- to medium-sized lymphocytes with nuclear irregularities were observed, and the CSF findings were consistent with stage IV malignant lymphoma. Flow cytometry did not detect any abnormal cell populations (Suppl. Data S1).

**Figure 3:**
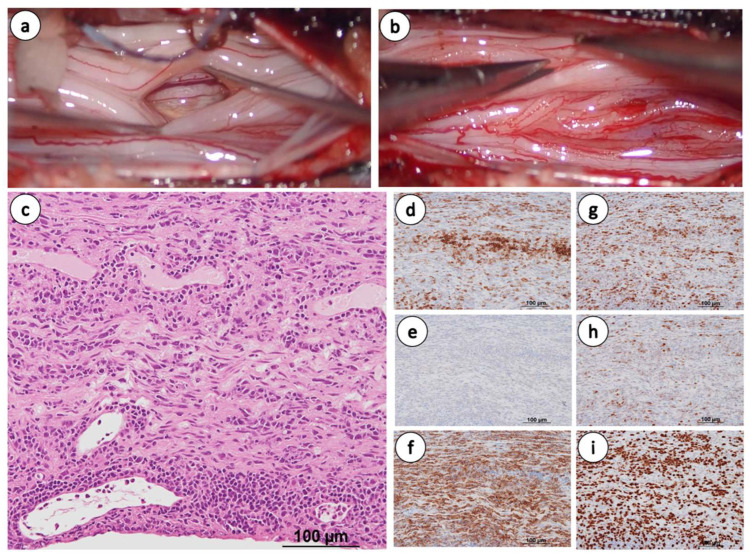
(**a**,**b**) Biopsy images. The cauda equina shows swelling and adhesions. (**c**–**i**) Histopathological examination of biopsy tissue. (**c**) Hematoxylin and eosin staining. (**d**) CD5 positive, (**e**) CD10 negative, (**f**) CD20 positive, (**g**) Bcl-6 positive, (**h**) MUM1 positive, and (**i**) Ki-67 index > 80%. The scale bar in the pathological image represents 100 µm.

To perform a biopsy, we resected the laminae from Th12 to L1, and incised the dural sac. No obvious tumor was found within the dural sac; however, the cauda equina showed diffuse erythema, swelling, and adhesions resulting in reduced mobility (Figure 3a,b). Histopathological examination revealed a diffuse infiltration of small- to medium-sized lymphocytes within the neural tissue (Figure 3c). Immunohistochemical staining was positive for CD5, CD20, Bcl-6, and MUM1, negative for CD10, and the Ki-67 index was >80% (Figure 3d–i), leading to the diagnosis of aggressive CD5-positive DLBCL of the non-GCB subtype. MYD88 mutation analysis was not performed in this case; however, the diagnosis of diffuse large B-cell lymphoma was established based on histopathological and immunophenotypic findings. The high proliferative index (Ki-67 > 80%) and immunophenotypic findings were inconsistent with indolent B-cell lymphoma, supporting the diagnosis of diffuse large B-cell lymphoma. Pre-treatment follow-up MRI revealed a new lesion in the left precentral gyrus, indicating additional CNS dissemination. At diagnosis, the patient was classified as Ann Arbor stage IV, with multiple extranodal lesions involving the brain and cauda equina. Further, the clinical parameters corresponded to a high-risk IPI category.

Table 1 summarizes the morphological, biochemical, and clinical risk factors identified in this case.

Following definitive diagnosis, the patient underwent five courses of immunochemotherapy with R-MPV in the hematology department. The administered doses were as follows: rituximab 375 mg/m^2^, methotrexate 2576 mg/m^2^, vincristine 1.0 mg/m^2^, and procarbazine 97 mg/m^2^. MTX and vincristine were initiated at 75% of the standard dose. Their doses were subsequently adjusted as appropriate based on age, adverse events, and serum drug concentrations. One month after treatment initiation, a reduction in the lesions was observed on gadolinium-enhanced T1-weighted MRI of the head and lumbar spine. After the completion of five courses of treatment, complete response (CR) was achieved. Lower extremity muscle strength improved, with MMT scores of 5/5 in the iliopsoas and quadriceps muscles bilaterally, 4/5 on the right side, and 3/5 on the left side for the tibialis anterior and gastrocnemius muscles. Subsequent chemotherapy was temporarily discontinued as the patient declined to undergo consolidation therapy. Approximately 4 months after achieving CR, nodular lesions reappeared in the left lateral ventricle on head CT and gadolinium-enhanced T1-weighted MR images of the head. Targeted therapy with tirabrutinib hydrochloride, a highly potent, selective, irreversible second-generation Bruton’s tyrosine kinase (BTK) inhibitor with CNS penetration, was initiated for the relapse of malignant lymphoma. Brain MR imaging showed further reduction in the lesion; however, the patient developed aspiration pneumonia and a urinary tract infection, leading to decreased oral intake and subsequent death.

## 3. Discussion

Primary CEL is extremely rare in patients with NL; to the best of our knowledge, only 29 cases have been reported in the English literature [1,4,5,6,7,8,9,10,11,12,13,14,15,16,17,18,19,20,21,22,23,24,25,26,27,28,29,30]. The clinical characteristics of these cases are summarized in Table 2. Our case was also included in Table 2. The age range was broad; from 11 to 80 years, with a mean age of 57.4 years and median age of 63.5 years, demonstrating a younger age at presentation compared to general NHL, which is consistent with the trends seen in PCNSL. The male-to-female ratio was 19:11, indicating a higher incidence in males. Symptoms at presentation included lower-extremity weakness in approximately 83% (25 cases), lower-extremity sensory disturbance in approximately 77% (23 cases), lumbosacral to lower-extremity pain in approximately 57% (17 cases), gait disturbance in approximately 43% (13 cases), and bladder/bowel dysfunction in approximately 33% (10 cases). Additionally, patients presented with headache, hearing loss, fever, sweating, tremors, and lethargy, showing various symptoms depending on the site of invasion. In all reviewed reports, a definitive diagnosis was established via histopathological examination; B-cell lymphoma accounted for approximately 86.2% of cases, with DLBCL identified as the predominant subtype. PET scanning was used as a supplementary imaging diagnostic tool in approximately 38% (11 cases) of the reports. Among these cases, FDG uptake was observed in 91% (10 cases) of patients who underwent PET imaging. It is generally known that most malignant lymphomas show strong FDG accumulation, and PET is also considered useful for detecting metastatic lesions [31]. In the present case, PET scanning could not be performed at our institution due to limited equipment accessibility, so alternative diagnostic tests were performed instead. However, in recent case reports of malignant lymphoma arising from the cauda equina, PET has been performed in many cases, and its implementation is considered desirable. The treatment modalities included combined chemotherapy and radiotherapy in 12 cases, chemotherapy alone in 10 cases, radiotherapy alone in two cases, combined chemotherapy and stem cell transplantation in one case, and corticosteroids alone in one case. In PCNSL, surgical intervention is generally limited to biopsy as the tumor is highly sensitive to chemotherapy and radiotherapy. Radiotherapy may be used as consolidation therapy, or in the relapse setting; however, its use in elderly patients is often carefully considered because of the risk of delayed neurotoxicity. In recent years, novel agents such as BTK inhibitors have emerged as promising therapeutic options for relapsed or refractory PCNSL [32]. Tumor size and lower extremity paralysis improvements with treatment were reported in 15 and 16 patients, respectively. At the final follow-up, 17 patients were confirmed to be alive, with a mean survival period of 20.4 months at the time of the case report.

The differential diagnosis includes metastatic tumors, inflammatory conditions such as sarcoidosis, and infectious diseases such as tuberculosis. These diseases can cause vertebral bone destruction or myelitis, presenting with pain and neurological disorders. However, histopathological examination is essential to achieve a definitive diagnosis of malignant lymphoma [33]. In our case, CSF showed an increase in cell count and protein concentration, as well as the presence of tumor cells. These findings are consistent with those of neurogenic malignant lymphoma; however, biopsy with histopathological examination is required to achieve a definitive diagnosis. A study by Suzuki et al. reported that primary CEL requires an average of approximately 6 months from symptom onset to diagnosis [1]. Delayed diagnosis can lead to the progression of neurological disorders, potentially leading to reduced mobility, a decline in quality of life and a poorer overall prognosis. Several studies have shown improvement in lower extremity paralysis with early diagnosis and treatment [1,4,6,8,9,12,13,14,15,16,17,18,21,22,26,29], and multiple reports recommend early neural biopsy [1,8,13].

The relative frequency of meningeal dissemination (MD) in PCNSL is 15.9 to 17.4%, indicating a low incidence. Brain parenchymal dissemination is particularly common among sites of meningeal dissemination, although the frequency of isolated meningeal and intraocular lymphomas is limited [34,35]. Among the 29 previously reported cases, brain lesions were documented in only three. In our case, although the initial brain MRI findings were negative, subsequent follow-up imaging identified the emergence of new brain lesions as the patient’s clinical workup continued. The prognostic impact of MD in primary CEL remains unclear; however, Kiewe et al. reported that MD did not affect the prognosis of patients [35]. Although brain MR revealed tumorous lesions within the brain parenchyma, immunochemotherapy with methotrexate (MTX) resulted in the regression of the brain parenchymal tumorous lesions.

Although radiotherapy has traditionally been considered the standard treatment for primary CEL, the addition of chemotherapy has significantly improved outcomes and overall prognosis. One prior study by Ferreri et al. reported that chemotherapy based on HD-MTX or HD-MTX combined with high-dose cytarabine (HD-ara-C) was effective for PCNSL treatment [36]. Among the 29 previously reported primary CEL cases, chemotherapy was utilized in 22 cases (Table 2), including MTX- or HD-MTX-containing regimens in 14 cases (64%). In our case, HD-MTX-based therapy was initially considered; however, because of impaired renal function, MTX had to be administered at a reduced dose. Five courses of immunochemotherapy with R-MPV were completed with this dose modification. Despite the reduced MTX dose, radiological regression of the brain and lumbar lesions was observed 1 month after treatment initiation, and a complete response was achieved upon completion of immunochemotherapy. These findings indicate that chemotherapy remains highly effective, and should be regarded as an important therapeutic option. Because a complete response was achieved, treatment beyond the fifth course of R-MPV was discontinued at the patient’s request. A recurrent brain lesion was detected four months after achieving CR; however, subsequent administration of tirabrutinib resulted in renewed tumor regression. Tirabrutinib was selected as the second-line treatment because the disease represented primary CNS lymphoma with cauda equina involvement, and BTK inhibitors are recognized as an effective salvage therapy for relapsed or refractory CNS lymphoma. Among the previously reported cases of primary cauda equina lymphoma, two were described as relapsed disease. Refractory disease is defined here as cases showing tumor progression or enlargement, two cases were considered refractory. Treatment for these cases consisted of combined chemotherapy and radiotherapy in one case, radiotherapy alone in one case, steroid alone in one case, and was not described in one case. However, therapeutic options for relapsed or refractory (r/r) disease remain limited and a significant clinical challenge. There was no description of second-line treatment at the time of relapse. In the present case, tirabrutinib, a highly selective BTK inhibitor with good central nervous system penetrability, was administered as second-line therapy after relapse following MTX-based immunochemotherapy. This case highlights the potential clinical relevance of BTK inhibition as a therapeutic option for relapsed or refractory lymphoma involving the cauda equina. While, no drugs are approved specifically for PCNSL in the United States or Europe, tirabrutinib received regulatory approval in Japan, Taiwan, and South Korea. The PROSPECT II study demonstrated its clinical efficacy in patients with r/r PCNSL, suggesting that BTK inhibition represents a promising therapeutic strategy for this disease. Therefore, the treatment strategy in this case was consistent with current clinical practice.

For radiotherapy of primary CEL, whole-brain radiotherapy including the posterior half of the globe is recommended for cases without ocular involvement, whereas whole-brain radiotherapy including the entire globe is recommended for cases with ocular involvement. However, in elderly patients, irradiation omission or dose reduction is performed to mitigate the risk of delayed neurotoxicity [32]. In our case, since immunochemotherapy was highly effective, combination with whole-brain radiotherapy including the posterior half of the globe at 23.4 Gy to 36 Gy was considered desirable as adjuvant therapy. However, this could not be performed because of worsening medical comorbidities and the lack of consent from the patient and family. Nevertheless, among the 29 previously reported cases, radiotherapy was selected for 14 cases, with particular efficacy demonstrated in 10 cases that combined chemotherapy and radiotherapy, suggesting that it should be considered as a treatment method

The biological and epigenomic features of primary cauda equina lymphoma remain poorly understood, and further investigation of the epigenomic landscape of B-cell lymphomas may provide novel therapeutic targets in the era of precision medicine [37].

## 4. Conclusions

Although diagnosis of primary CEL is difficult, early diagnosis and treatment are essential to optimize both neurological and vital prognoses. In cases presenting with neurological deficits of unknown etiology, intradural tumors should be considered in the differential diagnosis, and early neural biopsy should be considered.

## Figures and Tables

**Table 1:** Morphological, biochemical, and clinical risk factors in the present patient.

	Features	Findings in Present Case	Clinical Implication
Morphology	spinal lesion	cauda equina (T12–L1)	typical location of neurolymphomatosis
	histopathology	diffuse lymphocytic infiltration	confirms lymphoid neoplasm
	immunohistochemistry	CD5+, CD20+, Bcl6+, MUM1+	consistent with DLBCL
	proliferation index	Ki 67 > 80%	highly aggressive lymphoma
Biochemistry	serum biomarkers	elevated sIL-2R	suggestive of lymphoid malignancy
	CSF	pleocytosis and elevated protein with atypical lymphocytes, while glucose levels remained within the normal range.	suggestive of lymphomatous involvement of the CNS
Risk factors	IPI	high-risk category (age > 60, PS 2, stage IV, ≥2 extranodal sites)	indicates advanced disease and unfavorable prognosis
	Ann Arbor	stage IV	

**Table 2:** Summary of the clinical characteristics of past cases and the present case of primary cauda equina lymphoma.

Author	Pub Year	Age	Sex	Diagnosis	PET	Subtype	Treatment	F/U	Outcome	Post-Treatment		
										Relapsed	Tumor Size	Paralysis
Mauney et al. [30]	1983	68	F	SR	-	B-cell	RT	3	Alive	-	Unchanged	Unchanged
Toner et al. [29]	1989	59	M	Biopsy	-	B-cell	CT + RT	22	Alive	-	Reduction	Recovery
Klein et al. [28]	1990	29	F	SR	-	B-cell	ND	1.25	Dead	Brain	ND	Worse
Knopp et al. [27]	1994	69	F	Biopsy	-	ND	ND	ND	ND	-	ND	ND
Ooi et al. [26]	1996	16	M	Biopsy	-	T-cell	CT + RT	8	Dead	-	Growth	Recovery
Giobbia et al. [25]	1999	30	F	CSF	-	DLBCL	CT + RT	12	Alive	-	ND	ND
Zagami et al. [24]	2003	71	F	Biopsy	-	DLBCL	CT	15	Dead	-	ND	ND
Kumar et al. [23]	2005	60	M	Biopsy	-	LB	CT	6	Alive	-	ND	ND
Tajima et al. [22]	2007	67	F	Biopsy	-	DLBCL	CT + RT	36	Alive	-	Reduction	Recovery
Khong et al. [21]	2008	16	M	Biopsy	None	DLBCL	CT + RT	12	Alive	-	Reduction	Recovery
Morita et al. [20]	2009	67	M	SR	-	NK/T-cell	RT	14	Dead	Brain	Reduction	ND
Beitzke et al. [19]	2010	69	M	Biopsy	Uptake	DLBCL	CT	Soon	Dead	-	ND	ND
Teo et al. [18]	2012	58	M	Biopsy	-	DLBCL	CT + RT	24	Alive	-	ND	Recovery
Cugati et al. [17]	2012	11	M	SR	-	B-cell	CT + RT	12	Alive	-	Reduction	Recovery
Iwasaki et al. [16]	2012	69	M	Biopsy	-	DLBCL	CT + RT	18	Dead	-	Reduction	Recovery
Nishida et al. [15]	2012	47	M	CSF	-	DLBCL	CT + RT	18	Alive	-	Reduction	Recovery
Nakashima, et al. [14]	2014	59	M	Biopsy	Uptake	DLBCL	CT + RT	12	Alive	-	Reduction	Recovery
Broen et al. [13]	2014	75	F	Biopsy	-	DLBCL	Steroid	10	Dead	-	Growth	Worse
Broen et al. [13]	2014	71	F	Biopsy	Uptake	DLBCL	CT	ND	Alive	-	Reduction	Recovery
Shin et al. [12]	2016	79	F	Biopsy	-	DLBCL	CT	ND	Alive	-	Reduction	Recovery
Belcastro et al. [11]	2016	47	M	Biopsy	Uptake	DLBCL	CT	2	Dead	-	ND	ND
Wang et al. [10]	2016	65	M	CSF	Uptake	B-cell	ND	ND	ND	-	ND	ND
Geevarghese, et al. [9]	2017	46	M	SR	-	Follicular lymphoma	CT	30	Alive	-	ND	ND
Suzuki et al. [1]	2018	65	M	Biopsy	Uptake	DLBCL	CT	81	Alive	-	Reduction	Recovery
Sasaki et al. [8]	2018	62	M	Biopsy	Uptake	DLBCL	CT + RT	8	Alive	-	Reduction	Recovery
De Vries et al. [7]	2020	54	F	Biopsy	Uptake	T-cell LB	CT + RT	1.5	Dead	-	ND	ND
Kuhlman et al. [6]	2021	55	F	Biopsy	Uptake	DLBCL	CT/SCT	18	Alive	-	Reduction	Recovery
Suzuki et al. [5]	2023	80	M	Biopsy	Uptake	DLBCL	No	4	Dead	-	ND	ND
Lapolla et al. [4]	2024	77	M	SR	-	DLBCL	CT	12	Alive	-	Reduction	Recovery
Our case	2025	80	M	Biopsy	-	DLBCL	CT	6	Dead	Brain	Reduction	Recovery

F, female; M, male; Pub, publication; F/U, Follow-up (months); SR, surgical resection; DLBCL, diffuse large B-cell lymphoma; LB, lymphoblastic; RT, radiotherapy, CT, chemotherapy, ND, not described; SCT, stem cell transplantation; CSF, cerebrospinal fluid cytodiagnosis; No, no additional treatment.

## Data Availability

Data are contained within the article.

## Supplementary Materials

The following supporting information can be downloaded at: https://www.mdpi.com/article/10.3390/diseases14040129/s1, Supplemental Data S1: Flow cytometry using the LLA CD45 gating order set did not reveal any abnormal cell populations.
