# Supplementary material for: Diffuse Large B-Cell Lymphoma Arising from Cauda Equina: A Rare Case Report and Review of the Literature
Source: Diseases. 2026 Apr 2;14(4):129. doi: 10.3390/diseases14040129 (PMC13115372; doi:10.3390/diseases14040129)
Supplement: Supplementary file 1 [file diseases-14-00129-s001.zip › diseases-4168272-supplementary.pptx]

## Slide 1
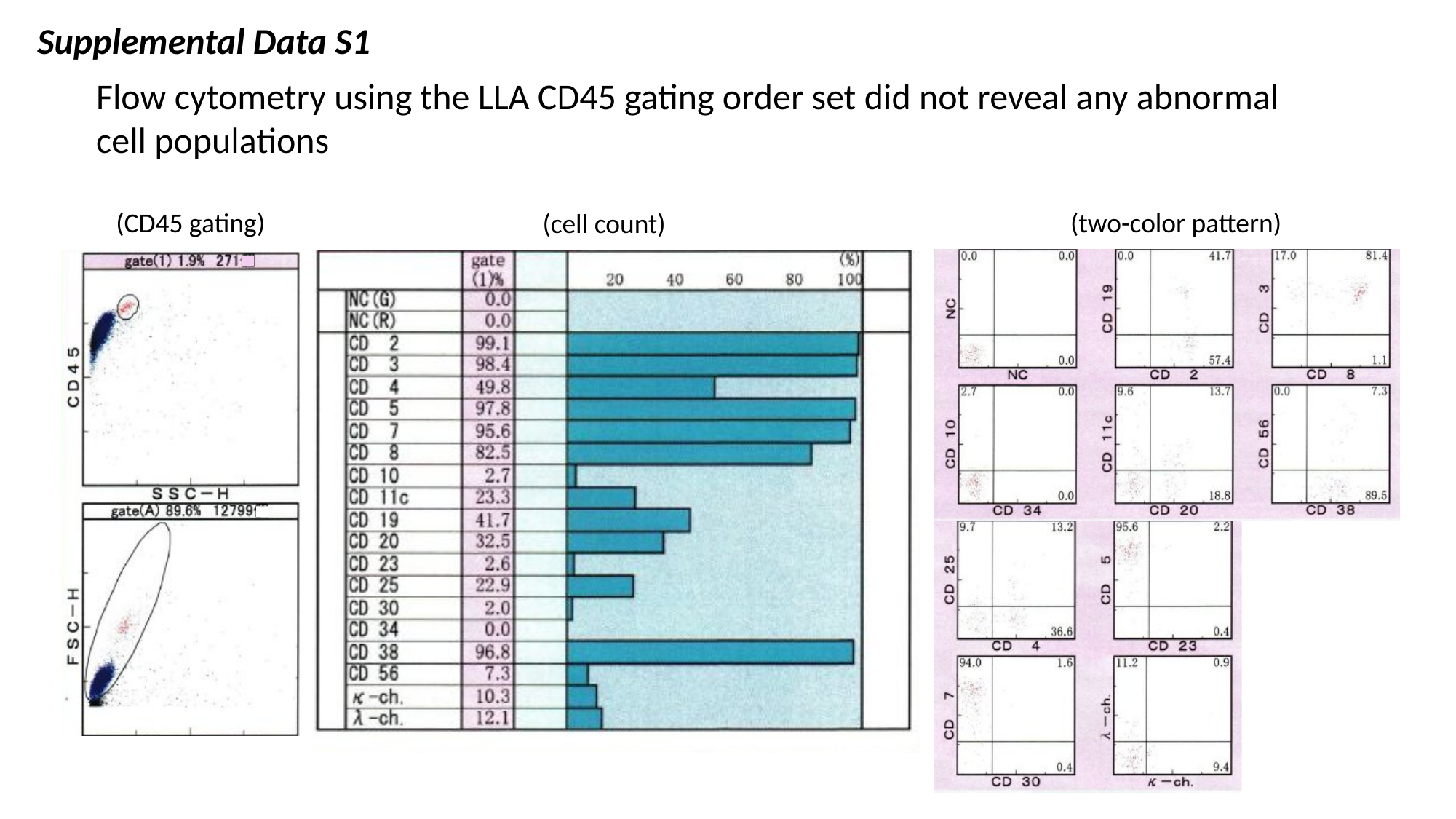

Supplemental Data S1
Flow cytometry using the LLA CD45 gating order set did not reveal any abnormal cell populations
(CD45 gating)
(two-color pattern)
(cell count)
